# Multiscreen to screen webinar for education beyond border: A review

**DOI:** 10.1016/j.amsu.2020.09.041

**Published:** 2020-10-09

**Authors:** Theddeus Octavianus Hari Prasetyono, Andreas Christian

**Affiliations:** a Division of Plastic Surgery, Department of Surgery Cipto Mangunkusumo Hospital/ Faculty of Medicine Universitas Indonesia, Jakarta, Indonesia; b ICTEC (Indonesian Clinical Training and Education Center), Cipto Mangunkusumo Hospital/ Faculty of Medicine Universitas Indonesia, Jakarta, Indonesia

**Keywords:** Distant education, Medical education, Internet, Pandemics, Social media

## Abstract

The COID-19 pandemic has forced people into a new way of adaptation with virtual meetings using videoconferencing apps.This study aims to report experiences of using a multiscreen to screen platform for sharing experiences in the form of Live Lecture and beyond. An observational study on distant CME events using multiscreen to screen webinar model was conducted from December 2019 - April 2020. Efficacy of the content delivery was measured using MCQs as pre- and post-test or by key questions. The videoconference was combined with a game platform to plant key points of the lecture. Among 68 webinars, there were 21 organied using multiscreen to screen platform, including 14 live lectures, 3 half-day webinars and 4 panel discussions. Only two live lectures were conducted with pre- and post-test. Six live lectures were added with key questions and game sessions. Time preference to oin the webinar session was at 9–10AM on the participants side; however, participations came from across the continents regardless the preference. Web-conference based activities become a new normal way of scientific meetings. A shorter event gets more participation and fewer number of participants leaving half-way through the event. Key questions and game sessions appear to be more interesting to the participants rather than pre- and post-test.

## Highlights

• COVID-19 pandemic compels health professionals to utilize teleteaching
• Exploring the optimal duration of webinars and its impact on participants' engagement.
• Analyze the best time zone to establish international webinars through experiences during the COVID-19 pandemic era.

## 1 Introduction

The current COVID-19 pandemic, which started off in Wuhan, China [1], has hit the world harshly and forced people to adapt quickly. People who lived far away from China were cautious whether their countries they live in would get the similar dynamics of the unprecedented outbreak. The story of people being resistant to the disease and rejected the measures to contain the virus were typical across the globe [2]. In the end, not less than 180 countries got hit and almost all the entire world was shutdown [3]. Measures to reduce and stop the virus transmission were massively implemented across the globe, which were expected to halt the outbreaks from further spreading [4]. After a while, it has started to look that no one could predict when the disaster would last for.

The effort to track people contacted prior to a positive COVID-19 subject is regarded as one of the important measures in the prevention and control scenario [5]. Finding new suspected, possible, probable and confirmed cases successfully which are subsequently followed with a strict protocol of quarantine and compliance would definitely help in containing the chain of infection. Realizing the significant impact it could have, private and government owned authorized bodies tried every effort possible, including the use of social media [1] and specifically crafted apps (applications) [6]. Older adults who complied to social distancing rule and self-quarantine have begun to utilize smartphone apps more to stay connected with families and friends [7]. These are only a few examples among the immense number of evidence showing people in the modern era have been using technology to adapt to the ever-changing life dynamics.

Apparently, social isolation as one of the few measures to contain the viral spread has put people away from organizing scientific meetings and congresses. Based on the senior author's personal observation and experiences, since February 2020 on ward, there had been many meetings and congresses postponed and aborted [8]. The postponement and cancellation of the meetings have caused damages with obvious schedule rearrangement, contracts renegotiation, loss of airfare and accommodation costs. These situations which so far came with uncertainties of when to end, require innovations and modifications of teaching, sharing, and learning experiences.

Interestingly, the COVID-19 pandemic has forced people into a new way of adaptation of replacing physical meetings with virtual meetings and persuade people to be more familiar with videoconferencing apps. It is not a surprise by then, to see the providers of videoconferencing services made a great success with their businesses only in a short time, especially in between March and April 2020 [9]. In an precedented occasion, the senior author has initiated to use a virtual business meeting platform since the December 2019 for scientific lecture sharing which appeared different from the traditional webinars. Traditional webinars appeared to be a single screen or very limited multiscreen meeting platform without allowing the speaker(s) to see the screen of an extraordinary number of participants. In short, the initiative was started earlier before it came up later as a booming virtual on-line scientific meeting which has been strongly influenced by the COVID-19 pandemic. This study aims to report experiences of using multi-screen to screen platforms for sharing experiences in the form of Live Lecture and beyond.

## 2 Points of view

An observational study based on experiences in organizing distant continuing medical education (CME) events using multiscreen to screen webinar model was conducted from December 2019–April 2020. The study does not need ethical approval. The platform for the distant meeting used was zoom (Zoom, San Jose California, USA). During the meeting, the host acts as the moderator of the room, while the faculties present their slides (PowerPoint or Keynote presentations or video demonstrations) from their own respective workplace or home. The host is the creator of the room and has the ability to mute participants and enable participants to unmute themselves. The host assigned 2 co-hosts to help maintain the order in the room by muting and unmuting participants while also being aware of any potential of distracting incidents. The role of the co-hosts is very important in large meetings, as people could be very unorderly and speak simultaneously during lecture and discussion.

The virtual waiting room was activated for every event. When the time for questions and answers (Q&A) session come, participants who would like to talk are advised to use the feature of “raise hand” by taking turns. Clicking the raise hand button allows the participants’ username to move up in the participants list; and a blue hand icon will appear beside their name. This would allow hosts or co-hosts to unmute the respective participants.

Similar to the traditional webinar, participants could also interact by texting in the chat box that is available by default. Although possible, participants were not advised to use the features to upload files/documents/pictures in the chat box; unless it was for sharing scientific evidence and permitted by the hosts in advance. For some reason, the feature of document upload is used for case discussion by invitation. The authorization to upload a file through the chat box could be enabled either only for hosts or all the participants.

Scientific meeting formats for CME may include but not limited to Live Lectures, Half-Day Webinar, Full-Day Webinar, WebDisc (web based-case discussion), Meet the Experts, Panel Discussion and Short Course. Presenters could use features of annotation besides having alternatives of using pointers. All formats allowed presenters to view the screens of the audiences, mimicking a real-life session in a physical room. Audiences could interact directly during the Q&A session, making the discussion interactive.

After scheduling the event, the person in charge of the event would usually contact the speakers or faculties several days or weeks before the event for an online technical briefing regarding the usage of the platform. Similar to the agreed time for the event with the faculties, the date of the technical meeting is made based on an agreeable time (zone) difference between the host and presenters.

The multiscreen to screen webinar was usually marketed or promoted through several social media platforms. Instagram (Facebook Inc., Menlo Park California, USA) is the main media to promote the event, besides Facebook (Facebook Inc., Menlo Park California, USA), institutional website, other social media groups and email. A digital flyer was created specifically for the event, with the date, time and topic of the presentation are shown.

The speakers’ photos, name and location were also printed in the digital flyer. The rundown was also described briefly in the flyer to highlight what topics would be discussed. A registration link was also stated in the flyer so that participants could share the event with other people. Contact information in the digital flyer might also be made available and the person in charge could guide the participants regarding the payment instructions if only the event is set not for free.

Efficacy of the content delivery was measured using multiple choice questions (MCQs) as pre- and post-test. The link for the pre-test using Google forms (Google LLC, Menlo Park, USA) was sent to the participants 2 h before the scheduled time, while the post-test was sent after the live lecture ended. Results were then tallied up and evaluated using *t*-test.

Moving onwards since April 7, 2020, the pre-and post-test format was changed into key questions, which were in the same MCQ format, but only sent to the participants right after the Q&A session. Subsequently, the rundown included a game which also works as an objective parameter to measure the efficacy. The game session does not only make the live lecture fun, but also to plant key points of the lecture to help participants memorize important points. Subsequently, a link of a feedback survey was sent over to participants’ emails.

Kahoot! apps (Kahoot! Oslo, Norway) was used to host the quizzes in the game session, and made a series of quizzes (open ended simple and short MCQs). The co-hosts shared the screen of the game via zoom, while the participants could play the game live from a separate tab in their own computer and gadget. For every quiz in the game session, participants would get direct feedback whether their answer to the quiz is correct or otherwise wrong. The game app comes with musical sound during the quizzes session and provides a sense of competition, whereby the names of the participants who answered correctly and faster would be mentioned from the first quiz to the last. At the end, it shows the first 5 winners.

## 3 Learning experiences

During the study period, there were 21 events that have been conducted using multiscreen to screen platforms among a total of 68 events when combined with the traditional webinars using different platforms. The events included 14 live lectures, 3 half-day webinars and 4 live panel discussions.

Topics of the webinars includes single area of discipline for live lectures and half-day webinars, while live panel discussions addressed general issues and topics related to the COVID-19. The last included research ethics during pandemic, surgical site infection, practice guidelines during pandemic and issue on tips and tricks in publication. The disciplines covered in the webinars are plastic, reconstructive and aesthetic surgery, hand surgery, microsurgery, orthopedic surgery, gynecology, and interventional radiology. In regards to the participants, they were mostly practitioners and trainees in respective disciplines, besides a smaller percentage of general practitioners and medical students. Participants came from all over the world and joined the events from their respective residence or places of preference.

Table 1 shows the summary of the webinars, including the average number of registrants, participants and duration of the webinar. The average number of participants who quit halftime for each type of webinar is also shown. Only webinars organized in late March and the whole month of April have the data on the registration number of each webinar.

**Table 1:** Summary of the webinars.

Type of Webinar	Number of scheduled webinars	Average number of registrants	Average number of participants	Average duration (minutes)	Average number of participants quitting halfway
Live lectures	15	321 (283–576) [n = 7^a^]	40 (16–339)	60 (60–120)	4 (0–98)
Half-day webinar	2	686 [n = 1^b^]	229 (55–403)	195 (180–210)	82 (21–142)
Panel discussion	4	101 [n = 1^c^]	57 (24–85)	60 (60–90)	9 (0–20)
Total	21				

a Only 7 out of 14 live lectures have information regarding the amount of registrants.

b Only 1 out of 3 Half-day webinar have information regarding the amount of registrants.

c Only 1 out of 4 Panel discussion have information regarding the amount of registrants.

The number of registrants is highest in half-day webinars, followed by live lectures and panel discussions. However, despite the higher number of registrants of half-day webinars, the average number of participants is higher in live lectures. The average number of participants for half-day webinars and panel discussions are roughly similar. Interestingly, the number of average participants quitting halftime is twice more in half-day webinars than in panel discussions. Besides heavily influenced by the popularity of the speakers and the topics, it looks that event duration plays a role. The shorter the event duration, the more the number of participations; and subsequently followed by the smaller number of people leaving before halftime.

The last six international live lectures were added with key questions and game sessions (Table 2). The data on the game session for the live lecture from Fortaleza, Brazil was not available due to technical obstacles. The obstacles were because we did not preannounce that it would be best for the participants to join the game from a separate tab or separate computer or gadget. Most of the participants were new to the game app and they failed to respond. Common problem of webinar was the same as other online learning events when the participants did not have optimal broadband connection.

**Table 2:** Summary of key questions and games in live lecture webinars.

Live lecture	Participants who participate in key questions	Average score (correct answers)	Participants who scored perfectly	Participants who participates in games	Participants who scored higher than 50%	Participant who scored perfectly
Fortaleza, Brazil (n = 124)	25 (20.2%)	2/3	6/25	no data available	no data available	no data available
Sao Paolo, Brazil (n = 276)	63 (22.8%)	2/3	28/63	45 (16.3%)	13/45	2/45
Atlanta, Georgia, USA (n = 166)	29 (17.5%)	3/5	2/29	25 (15%)	6/25	3/25
El Paso, Texas, USA (n = 301)	22 (7.3%)	2/3	8/22	30 (10%)	4/30	1/30
Milan, Italy (n = 339)	44 (13%)	2/3	23/44	62 (18.3%)	39/62	16/62
Singapore & Jakarta (n = 198)	57 (28.8%)	2/3	30/57	54 (27.3%)	23/54	4/54

Only two live lectures were conducted with a pre- and post-test. Although the post-test score increased when compared to the pretest score, Table 3 shows insignificant difference. Besides, only a few participants took part in both pre- and post-tests. It looks that participants lacked interest in joining pre-and post-test. As most of the events were organized without payment needed for participants to register, the participants did not feel obliged to sit for the pre- and post-test. If it were mandated, the pre-and post-test would work. The modification of testing the efficacy of scientific content delivery through key questions looked to be more interesting to the participants. They did not feel being measured, rather assessed themselves voluntarily.

**Table 3:** Summary of live lectures with pre- and post-tests.

Live lecture	Number of participation in both pre-test and post-test	Average pre-test score	Average post-test score	p value
Jakarta, Indonesia 1	7/18	41.4 ± SD 23.4	60 (60–90)	p = 0.061^a^
Jakarta, Indonesia 2	6/25	50 ± SD 14.1	61.7 ± SD 28.6	p = 0.443^b^

a Wilcoxon sign rank test.

b paired *t*-test.

**Fig. 1:**
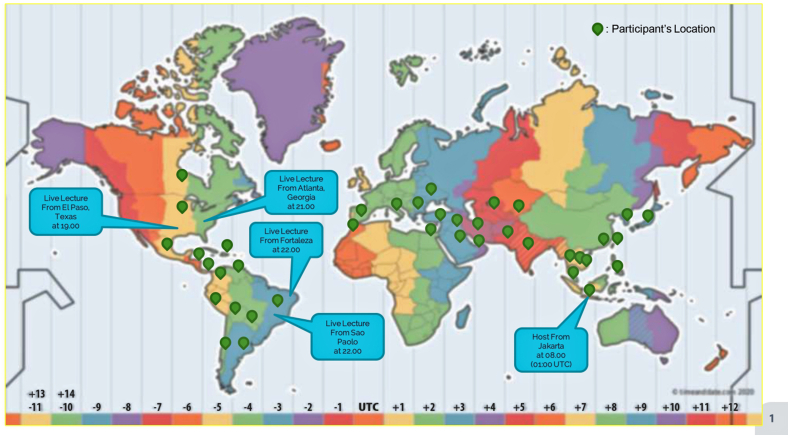
Map of time zone of different live lectures. The host resided in Jakarta, Indonesia, which is located at GMT+7 (Greenwich Mean Time) zone. Evening lectures from American continent were followed by participants from across the world.

Based on the feedback given by 224 participants of 7 international events, 29% respondents preferred to join webinars at 9.00–10.00AM in their respective time zones; followed by 12.00–1.00PM (25.9%), 6.00–7.00PM (21.4%) and 4.00–5.00PM (17.4%). Most of the respondents agreed that a webinar session after 7.00PM is not ideal. Fig. 1 specifically shows specific lectures from American continent, which were attracting participants from all 5 continents.

The feedback about time preference to join webinar is a good guide for people to organize webinar, although there is no ideal time for all. If only participant population target is clearly limited, then the time should be fit accordingly into 9.00–10.00AM or 12.00–1.00 PM at the participants’ side. However, people who are interested in the topics and presenters would still join the webinar regardless of the time preference (Fig. 1). Moreover, overall satisfaction level is regarded as high by participants. One of the influencing factors might refer to the free registration for all webinars. It is not clear yet whether the satisfaction level would decrease when the webinar comes with registration fee. This would need more observation and potential study when the pandemic is over to allow a more balanced situation; because during the pandemic, it seems that the international world accepts the paradigm that free webinars are taken for granted.

A data set taken from 11 webinars (with 389 respondents) revealed 35% participants got their information from Instagram and 33% from friends/colleagues. This is subsequently followed by email (16%) and department or workplace unit (9%). The rest of the participants got information from WhatsApp (4%), browsing (3%), and Telegram (1%). Overall, participants were satisfied with the program sessions provided. Using Likert scale from 1 to 5 with which 1 represents the least and 5 represents the most, participants rated 4 to 5 for all questions. The satisfaction included “time deliverance of the topic was enough,” “lecturer has good understanding of the topic discussed,” “lecturer answered the questions well and detail,” “the topic discussed was relevant to your practice,” and “the time for questions and answers session is good and enough.” (Table is presented as supplementary material.)

## 4 Discussion

The prohibitive meetings during the pandemic have forced people into an undeniably fast live adaptation in which people suddenly became familiarized themselves with web-conferencing based activities. Video conferencing became common for scientific sharing and discussion, which in the past it was used mostly for business purposes or just for fun activities. Webinar became much more popular in a sudden. Without disregarding the influencing factors for effective learning, webinar has been used to transfer knowledge adequately [10,11]. In traditional model, speakers and moderator are positioned in the same room and usually not able to see participants by face in an extraordinarily large number of gadget and computer's screen [12]. The idea of using the multiscreen to screen platform for scientific webinars is considered new; only recently this model have been practiced by scientific communities all over the world. To the best of the authors' knowledge, this study is the largest series of webinars conducted by a single unit in a relatively short time period (68 webinars in 5 months) reported in the literature [13].

Without underestimating the very great extent of online education in general, it looked that webinar has been a vast mode of knowledge transfer in medicine, especially during the pandemic [13,14]. This is true in regard to continuing medical education. In other scientific disciplines, such as political science, scholars seem to be still reluctant in embracing online tools. Traditional tools of scholarship are still in their preference, although not for the graduates [15]. However, it shows in a mixed method study that trainees, who are considered as young generation, had high satisfaction level and preferred greater levels learner-teacher interaction.[16] Besides, the study shows similar outcome with our observation that webinar is ideal if it is conducted for less than 90 min.^16^

Table 2 shows that the participation in the key questions and game sessions is roughly similar. Obviously, it is difficult to analyze the results of the number of participants who scored perfectly and more than half correct in both key questions and game sessions due to many influencing factors to be considered. This would be an interesting topic to study through a well-designed prospective research in the future. Nevertheless, this study is the first one reporting combined game and multiscreen to screen platforms for scientific virtual meeting to facilitate the audience assess their own receptive knowledge.

Virtual panel sessions discussing national guidelines related to the COVID-19 pandemic and its aftermath were also held through this platform besides other topics. When it comes with questions that require heavy elaboration, direct interaction between faculties and participants could be more effective than typing questions through the chat box. Annotations could also be done during screen sharing, which makes the discussion more enjoyable.

A webinar in general is organized using a platform that is equipped with a recording system. Recording could be set automatically or manually; and also stored in a cloud system or in the computer of the host. Technically, recorded video is easily made available for all learning sessions with or without additional cost. Last but not least, multiscreen to screen platform for webinar is also potentially elaborated as a platform for virtual scientific congress, which would need multiroom to organize simultaneous sessions. And the recorded system would also be good to facilitate continuing education in a much less mobilisation of resources by embedding the recordings into any platform for offline viewing.

In summary, web-conference based activities become a new normal way of scientific meetings. A shorter event gets more participation and fewer number of participants leaving half-way through the event. Key questions and game sessions appear to be more interesting to the participants rather than pre- and post-test. A prospective study on the relation of self-assessment model and all the influential factors would be a topic for future study.

## Guarantor

Author Theddeus O.H. Prasetyono and Co-author Andreas Christian is the guarantor of this research project.

## Registration of research studies

1. Name of the registry:
2. Unique Identifying number or registration ID:
3. Hyperlink to your specific registration (must be publicly accessible and will be checked):

## Funding

Not available.

## Ethical approval

This research does not involve patients requiring ethical approval.

## Consent

The feedback form that were obtained after each webinar session was sent to each participant's email voluntarily; participants' names, initials were not included in the questionnaire sent to their emails. Hence, no privacy has been breached through this study.

## Author contributions

Theddeus O.H. Prasetyono contributed to this article in creating the conception and design of the study, conducting the acquisition and performing the analysis and interpretation of the data, writing the manuscript draft and generating the final approval of the manuscript to be submitted.

Andreas Christian contributed to this article in conducting the acquisition and performing the analysis and interpretation of the data and writing the manuscript draft.

## Declaration of competing interest

The authors report no conflict of interest.
