# Supplementary material for: Multiscreen to screen webinar for education beyond border: A review
Source: Ann Med Surg (Lond). 2020 Oct 9;59:237–41. doi: 10.1016/j.amsu.2020.09.041 (PMC7546648; doi:10.1016/j.amsu.2020.09.041)
Supplement: Multimedia component 1 [file mmc1.docx]

Supplementary meaterials

Table. Average of participants’ satisfaction using Likert scale

| **Webinars** | **Time deliverance of topic is enough** | **Lecturer have a good understanding of the topic discussed** | **Lecturer answered the questions well and in detail** | **The topic discussed is relevant to your clinical practice** | **The time for questions and answers session is good and enough** |
| --- | --- | --- | --- | --- | --- |
| Fortaleza, Brazil  (n=14) | 4 (3-5) | 4.5 (4-5) | 4 (4-5) | 4 (3-5) | 4 (2-5) |
| Jakarta, Indonesia  (n=4) | 4.50 (4-5) | 4.5 (4-5) | 4.50 (4-5) | 4.50 (4-5) | 4.50 (4-5) |
| Jakarta, Indonesia  (n=4) | 3.75 ± SD 0.96 | 4 ± SD 0.82 | 4 ± SD 0.82 | 4 ± SD 0.82 | 4 ± SD 0.82 |
| Sao Paolo, Brazil  (n=30) | 4 (2-5) | 4 (2-5) | 4 (2-5) | 4 (2-5) | 4 (2-5) |
| Atlanta, Georgia, USA  (n= 20) | 4 (2-5) | 4 (2-5) | 4 (2-5) | 4 (3-5) | 4.5 (2-5) |
| El Paso, Texas, USA  (n= 27) | 5 (3-5) | 5 (3-5) | 5 (3-5) | 5 (3-5) | 5 (3-5) |
| Jakarta, Indonesia*  (n=16) | 4 (1-5) | 4 (3-5) | 4 (3-5) | 4 (2-5) | 4 (3-5) |
| Singapore. Biskek, Kyrgyztan. Seoul, Korea**  (n=60) | 5 (2-5) | 5 (2-5) | 5 (2-5) | 5 (3-5) | 5 (3-5) |
| Jakarta, Indonesia  (n=47) | 5 (1-5) | 5 (2-5) | 5 (2-5) | 5 (3-5) | 5 (1-5) |
| Milan, Italy  (n= 48) | 5 (1-5) | 5 (2-5) | 5 (2-5) | 5 (2-5) | 4 (3-5) |
| Singapore & Jakarta, Indonesia  (n= 31) | 4 (2-5) | 4 (2-5) | 4 (2-5) | 4 (2-5) | 4 (3-5) |
| Total Average  (n=301) | 4 (1-5) | 5 (2-5) | 5 (2-5) | 5 (2-5) | 4 (1-5) |
| Overall Average | 5 (1-5) | | | | |

* Panel discussion; ** Half-day webinar; Likert scale 1 represents the least, 5 represents the most
